# *Rhodococcus equi*’s Extreme Resistance to Hydrogen Peroxide Is Mainly Conferred by One of Its Four Catalase Genes

**DOI:** 10.1371/journal.pone.0042396

**Published:** 2012-08-06

**Authors:** Pauline Bidaud, Laurent Hébert, Corinne Barbey, Anne-Cécile Appourchaux, Riccardo Torelli, Maurizio Sanguinetti, Claire Laugier, Sandrine Petry

**Affiliations:** 1 Dozulé Laboratory for Equine Diseases, Unit Bacteriology and Parasitology, ANSES, Goustranville, France; 2 Institute of Microbiology, Catholic University of Sacred Heart, Rome, Italy; East Carolina University School of Medicine, United States of America

## Abstract

*Rhodococcus equi* is one of the most widespread causes of disease in foals aged from 1 to 6 months. *R. equi* possesses antioxidant defense mechanisms to protect it from reactive oxygen metabolites such as hydrogen peroxide (H_2_O_2_) generated during the respiratory burst of phagocytic cells. These defense mechanisms include enzymes such as catalase, which detoxify hydrogen peroxide. Recently, an analysis of the *R. equi* 103 genome sequence revealed the presence of four potential catalase genes. We first constructed Δ*katA-*, Δ*katB-*, Δ*katC-*and Δ*katD* **-**deficient mutants to study the ability of *R. equi* to survive exposure to H_2_O_2_ *in vitro* and within mouse peritoneal macrophages. Results showed that Δ*katA* and, to a lesser extent Δ*katC,* were affected by 80 mM H_2_O_2_. Moreover, *katA* deletion seems to significantly affect the ability of *R. equi* to survive within murine macrophages. We finally investigated the expression of the four catalases in response to H_2_O_2_ assays with a real time PCR technique. Results showed that *katA* is overexpressed 367.9 times (±122.6) in response to exposure to 50 mM of H_2_O_2_ added in the stationary phase, and 3.11 times (±0.59) when treatment was administered in the exponential phase. In untreated bacteria, *katB*, *katC* and *katD* were overexpressed from 4.3 to 17.5 times in the stationary compared to the exponential phase. Taken together, our results show that KatA is the major catalase involved in the extreme H_2_O_2_ resistance capability of *R. equi*.

## Introduction

*Rhodococcus equi* is a Gram-positive, facultative intracellular pathogen affecting foals up to six months of age. The major manifestation of *R. equi* infection is chronic suppurative bronchopneumonia, but 50% of contaminated foals also show clinical signs of intestinal disease and occasionally septic arthritis or osteomyelitis [1]. It is now well known that, in addition to being a veterinary concern, *R. equi* is an important opportunistic zoonotic pathogen mainly identified in immunocompromised people, and particularly AIDS patients [2], [3]. Interestingly, different cases of infection by *R. equi* have also been reported in immunocompetent patients [4], [5].

*R. equi* pathogenicity is closely linked to the bacterium’s ability to survive and replicate in the lung macrophages of infected foals [6], where bacteria are generally killed by the combined action of a low pH (4.0–5.0), hydrolytic enzymes (including proteases, lipases, DNAses and RNAses) and the production of reactive oxygen metabolites such as hydrogen peroxide, H_2_O_2_ [7]. *R. equi* has been characterized as being able to survive and multiply within a membrane-bound vacuole inside macrophages. This capability is conferred by the presence of a 80–90 kb virulence plasmid which inhibits i) the maturation of phagosomes, leading to the absence of phagosome acidification and prevents ii) the fusion of phagosomes with the lysosomes containing many hydrolytic enzymes [7]. In addition, *R. equi* must be able to mount an antioxidant defense system to ensure its persistence and multiplication within the highly oxidative environment of host macrophages generated notably by the reactive oxygen species (ROS) producing phagocyte oxidase Nox2 [8]. It has already been shown that *R. equi* is highly resistant to the action of H_2_O_2_ [9], though plasmid-encoded proteins do not play a role in *R. equi*’s resistance to H_2_O_2_ [9]. An analysis of the *R. equi* 103 genome revealed the presence of numerous genes involved in oxidative stress resistance. Four genes in particular display homology with catalases [10], which are enzymes responsible for detoxifying H_2_O_2_ [11].

This was the background for our investigation into the causes of the extreme resistance of *R. equi* to oxidative stress. Our study therefore focused on an analysis of the four potential catalase genes *katA*, *katB*, *katC* and *katD*. We studied the role of these proteins in H_2_O_2_ resistance and in the ability of *R. equi* to survive within mouse peritoneal macrophages. These results were confirmed with the expression analysis of the catalase genes in response to H_2_O_2_ exposure.

## Results

### Identification of Four Genes Potentially Involved in H_2_O_2_ Resistance

**Figure 1:**
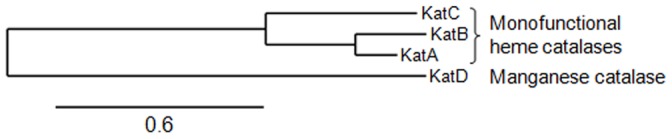
Phylogenetic relationships between the catalases of *Rhodococcus equi* 103. Catalases were aligned using Phylogeny.fr (http://www.phylogeny.fr/) [37].

The *in silico* analysis of the genome sequence of the *R. equi* 103 strain [10] revealed the presence of four potential catalase genes, designated *katA*, *katB*, *katC* and *katD*. These four genes are at separate locations on the chromosome and do not seem to share genetic links. Sequence analysis of these proteins shows that KatA, KatB and KatC share the conserved catalytic residues and metal ligand amino acids of members of monofunctional heme catalases [12]. KatD shares the conserved catalytic residues and metal ligand amino acids of members of the nonheme catalases. These nonheme catalases utilize manganese ions instead of ferric heme in their active site and are therefore also known as manganese catalases [13]. No catalase of the bifunctional catalase-peroxidase family was identified. Phylogenetic relationships between the four catalases are represented in Figure 1, which shows that the three heme and manganese catalases constitute a distinct group, and that KatA and KatB are closely related. No signal sequence and/or transmembrane domain were identified for these four catalases by TMHMM [14] or SignalP [15] analysis.

In order to estimate the involvement of each catalase in *R. equi’*s oxidative stress resistance, we constructed corresponding mutants. To define the physiological roles of the four catalases, we studied the growth characteristics of the four deficient mutants. The characteristics were the same as those of the wild type (WT) strain under aerobic growth in BHI medium (data not shown). This similarity demonstrates that the catalases are not essential to cell viability under aerobic conditions.

### Δ*katA* and Δ*katC* are the most Sensitive to H_2_O_2_ Treatment

**Figure 2:**
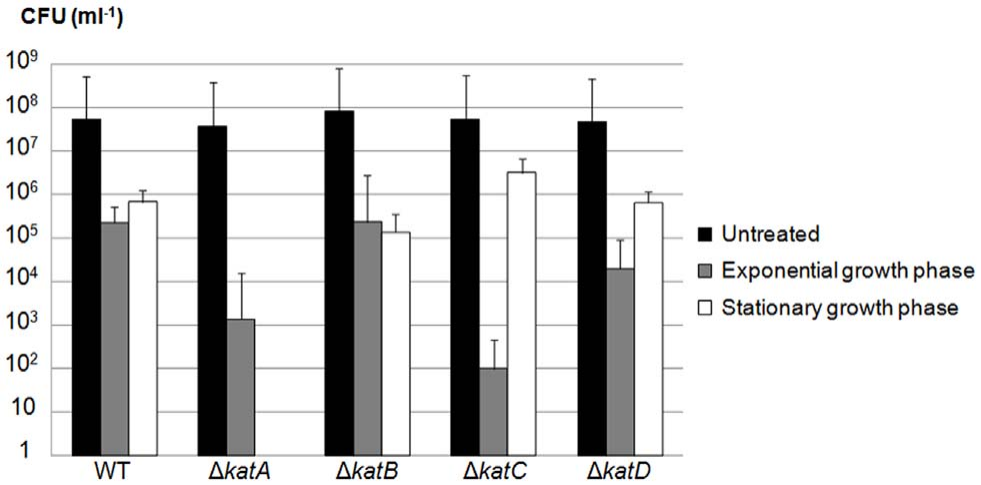
Decrease in Δ*katA* and Δ*katC* survival after exposure to H_2_O_2_ (80 mM, 30 min). *R. equi* WT, Δ*katA*, Δ*katB*, Δ*katC* or Δ*katD* were grown to exponential (OD_600_ = 0.2) and stationary growth phases (16 h of growth). The number of bacterial cells was standardized by dilution of stationary phase cells before treatment. Cells were challenged with 80 mM of H_2_O_2_ for 30 min under agitation. Viability was assayed both before (black bars) and after (grey and white bars) H_2_O_2_ treatment by plating the bacterial cells on BHI agar. The mean values of three independent experiments are represented, and the standard deviations are indicated.

To evaluate the effect of exogenous H_2_O_2_ stress on the survival of each mutant, cells were challenged with 80 mM of H_2_O_2_ under agitation, and CFUs determined 30 min after treatment (Figure 2). Δ*katA* was the most sensitive mutant to H_2_O_2_ since it did not survive when treated in the stationary phase, and its survival decreased by two orders of magnitude compared to the WT strain when treated in the exponential phase. Δ*katC* survived as well as the WT strain in the stationary phase but in contrast, when the cells were in the exponential phase, Δ*katC* was the most susceptible to H_2_O_2_ exposure with a decrease in survival of over three magnitudes compared to the WT strain. Both Δ*katB* and Δ*katD* had the same resistance capability as the WT strain, whether in exponential or stationary phases. The drop in survival rates for Δ*katA* and Δ*katC* after exposure to H_2_O_2_ demonstrate that, in these experimental conditions, only KatA and KatC primarily contribute to the oxidative defense system of *R. equi*.

### Δ*katA* is the most Susceptible to Elimination by Macrophages

**Figure 3:**
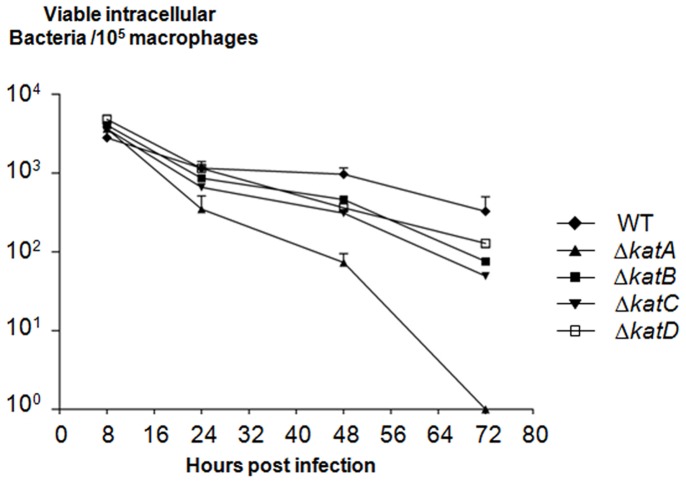
Δ*katA* is the most susceptible to elimination by macrophages. Mice were infected with 10^7^ to 10^8^ cells of *R. equi* WT, Δ*katA*, Δ*katB*, Δ*katC* or Δ*katD* by intraperitoneal injection. Intramacrophages bacteria viability was evaluated 24-, 48-and 72 h post infection. The data are the mean numbers of viable intracellular bacteria per 10^5^ macrophages ± standard deviations (error bars) for three independent experiments with three wells in each experiment.

During phagocytosis, phagocytic cells generate superoxide and other ROS involved in antibacterial activity [16]. This study compared the intracellular survival of the WT and Δ*katA*, Δ*katB*, Δ*katC* and Δ*katD* strains inside infected mouse peritoneal macrophages (Figure 3). No significant difference was observed in the levels of *R. equi* strains recovered 8 h post infection, suggesting that the different strains possessed similar abilities to infect macrophages (Figure 3). Δ*katA* was shown to be the most susceptible mutant, completely eliminated by macrophages 72 h post infection (*P*<0.05). Δ*katB*, Δ*katC* and Δ*katD* were shown to be more susceptible than the WT strain to macrophage killing at 48-and 72-h time points (P<0.05) but were not completely eliminated from macrophages 72 h post infection. The survival rate of each strain was similar by 24 h post infection but the WT strain’s ability to survive intracellularly was greater than that of Δ*katB*, Δ*katC* and Δ*katD* at 48-and 72-h time points (*P*<0.05). The inability of Δ*katA* to survive in macrophages beyond 72 h reveals KatA’s involvement in the intramacrophage resistance of *R. equi* to H_2_O_2._

### *katA* is Overexpressed in Response to H_2_O_2_ Treatment

**Figure 4:**
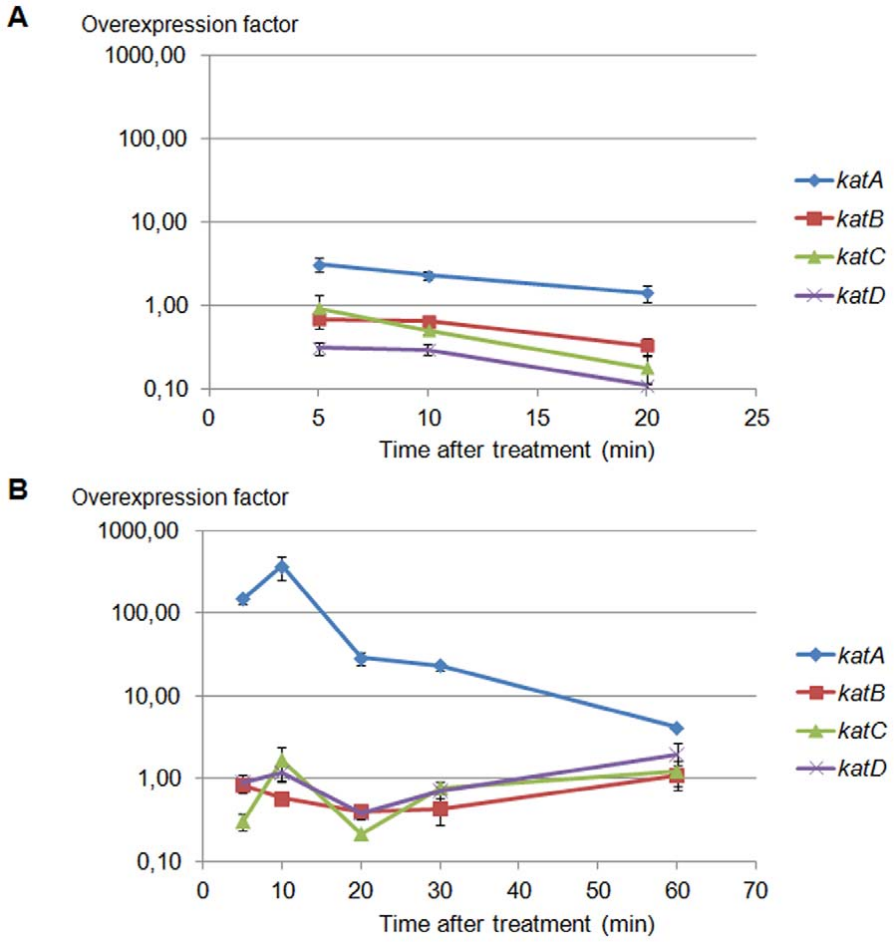
*katA* is overexpressed after H_2_O_2_ treatment (50 mM). *R. equi* WT was treated with 50 mM H_2_O_2_ in (A) the exponential phase (OD_600_ = 0.4) or (B) the stationary phase. cDNAs derived from total RNA were used for real time PCR. The overexpression factor in treated bacteria *vs* untreated bacteria, was calculated using the 2^−ΔΔCt^ method [36]. The data are the mean of overexpression factor ± standard deviation (error bars) of triplicate measurements from four reverse transcriptions of two independent experiments. To evaluate overexpressions in the exponential phase (A), we only considered time points 5, 10 and 20 min post exposure to avoid any effects of bacterial transition in the stationary growth phase.

We evaluated the effect of a sublethal H_2_O_2_ concentration (50 mM) defined previously (data not shown) on the expression of *katA*, *katB*, *katC* and *katD* transcripts during exponential (Figure 4A) and stationary phases (Figure 4B). The expression of catalases was measured at several timepoints: 5, 10 and 20 minutes after treatment in the exponential phase and 5, 10, 20, 30 and 60 minutes after treatment in the stationary phase. Results showed that only the *katA* gene was overexpressed in response to H_2_O_2_ treatment. For example, when H_2_O_2_ was added in the exponential phase, *katA* was overexpressed 3.11 (±0.59) times in bacteria treated for 5 min compared to untreated bacteria (Figure 4A). However, when H_2_O_2_ was added in the stationary phase, *katA* was overexpressed 367.90 (±122.63) times in bacteria treated for 10 min compared to untreated bacteria (Figure 4B). We did not observe any modifications in the rate of transcripts for *katB*, *katC* or *katD* (Figures 4A and 4B).

### *katB*, *katC* and *katD* are Overexpressed in the Stationary Growth Phase Compared to the Exponential Phase

**Figure 5:**
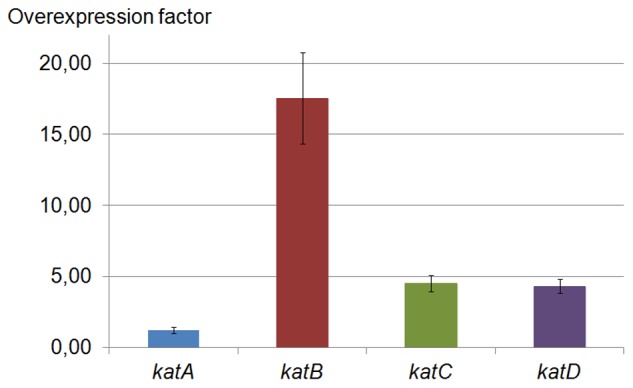
In untreated bacteria, *katB*, *katC* and *katD* are overexpressed in the stationary growth phase. *R. equi* WT was grown in BHI broth without any H_2_O_2_ treatment and cells were collected at the exponential (OD of 0.4) and stationary (OD of 1) growth phases for total RNA extraction. cDNAs derived from total RNA, were used for real time PCR. The overexpression factor was calculated using the 2^−ΔΔCt^ method [36]. The data are the mean of overexpression factor in stationary phase collected bacteria compared to exponential phase collected bacteria ± standard deviation of triplicate measurements from four reverse transcriptions of two independent experiments.

In untreated bacteria, we observed overexpressions of *katB*, *katC* and *katD* in the stationary phase compared to the exponential phase. Irrespective of exposure to oxidative stress, *katB* was overexpressed 17.55 (±3.22) times in the stationary phase compared to the exponential phase (Figure 5). *katC* and *katD* were overexpressed 4.51 (±0.59) and 4.31 (±0.50) times in the stationary phase compared to the exponential phase, respectively (Figure 5).

## Discussion

Resistance to oxidative stress is one of the key processes that allow pathogens to survive within macrophages, which is why pathogenic bacteria have developed different antioxidative defenses to eliminate ROS or repair their damage [17]. *In silico* analysis shows that *R. equi* 103 contains three monofunctional heme catalases (KatA, KatB and KatC) and a monofunctional manganese catalase (KatD). The present study addressed the issue of the involvement of these potential H_2_O_2_-degrading catalase enzymes in *R. equi* virulence and demonstrates that survival following exposure to exogenous H_2_O_2_ and the efficient proliferation of this bacterium in macrophages mainly rely on the activities of KatA, one of its four catalases.

Benoit *et al.* showed that the extreme resistance of *R. equi* to exposure to a high H_2_O_2_ concentration (100 mM) did not depend on plasmid-encoded proteins [9]. This was confirmed by the analysis of virulence plasmid–chromosome regulatory crosstalk by Letek *et al.* [10] which shows that the presence of the virulence plasmid did not interfere with the expression of catalase genes. These results suggest that chromosomal genes are key to this function, and genomic analysis of the *R. equi* chromosome identified an unusually high number of catalases: three monofunctional heme catalases (*katA*, *katB* and *katC*) and one manganese catalase (*katD*). Surprisingly, no bifunctional catalase-peroxidase was identified although for numerous pathogens, these enzymes have already been shown to be involved in the infection of macrophages [18]–[20].

An analysis of the physiological functions of the four catalases following exposure to exogenously-provided H_2_O_2_ showed that only KatA and KatC are part of the peroxide defense system of *R. equi*. KatA seemed to be more important than KatC under these stress conditions. The fact that *katB* and *katD* mutations did not affect survival following H_2_O_2_ exposure suggests that either i) these catalases are non functional, ii) they function as a surrogate in the absence of the primary antioxidant, but their role can only be detected in a double or triple catalase mutant [21] or iii) they have specialized roles for protecting cells against oxidative stress perhaps due to different cellular locations. Although no secretion peptide signal was identified for *R. equi* catalases, it has already been shown that a catalase from *B. subtilis* could be secreted independently of the Sec-secretion apparatus [22], [23].

Inactivation of catalase-encoding genes had little if any effect on pathogenicity-associated traits of numerous bacteria including *Salmonella typhimurium* [24], *Yersinia pestis* [25], *Staphylococcus aureus* [26], and *Neisseria gonorrhoeae* [27]. This research demonstrates that significantly lower quantities of viable intracellular bacteria were found in the infected murine macrophages of *ΔkatA*-deficient mutants during the 72-h infection period than in the WT strain. This suggests that the *katA* gene is linked to *R. equi* virulence mechanisms. This observation could be linked to the oxidative microenvironment present within phagosomes, where ROS and reactive nitrogen species are synthesized in huge quantities following macrophage phagocytosis [17], [28].

Studies of physiological functions are complex when multiple catalases are present in a single bacterial cell and differences in regulation are a critical factor in understanding the *in vivo* roles of these enzymes. Our results show that only *katA* expression is induced in response to exogenous H_2_O_2_. This fast and transitory overexpression is induced mainly in the stationary phase (overexpression factor of 367.9±122.6), which is in accordance with the critical role of *katA* in stationary**-**phase survival. In contrast, despite its role in the exponential phase, *katC* is not induced in the stationary phase. This phenomenon, already observed for a catalase-peroxidase of *Legionella pneumophila* [11], is not yet fully understood. In untreated bacteria, *katB*, *katC* and *katD* expressions increase in the stationary growth phase. These overexpressions could prepare the cells for subsequent stresses [29]. In this context, we could hypothesize that the lack of effect of the mutation of these genes in the stationary phase is due to a compensatory catalase mechanism between them or that these catalases play a role in protecting against the oxidative stress generated by growth in a minimal medium as already described [30]. Our results therefore suggest that *katA* is regulated by a specific stress response transcriptional regulator able to sense the presence of H_2_O_2_, whereas the other three catalases are under the control of a RpoS-like central regulator of gene expression in the stationary phase [31]. Moreover, other unidentified conditions could induce *katB*, *katC* and *katD* expression. We could suggest, for example, that the expression of manganese catalase *katD* is induced by iron starvation as previously described for the manganese catalase of *Pseudomonas aeruginosa* [32].

### Conclusions

This study addressed two fundamental questions: how are the four *R. equi* catalases involved in survival i) following external exposure to H_2_O_2_ and ii) in mouse peritoneal macrophages? Taken together, our results show that KatA is the major determinant of resistance to exogenous H_2_O_2_ exposure in the stationary phase and, to a lesser extent, in the exponential phase. KatA is also crucial to *R. equi*’s survival in mouse peritoneal macrophages. Of the other three *R. equi* catalases, only KatC plays a role in resistance to external H_2_O_2_ exposure, but only in the exponential phase.

## Materials and Methods

### Ethics Statement

The animal experiments (Survival assays in mouse peritoneal macrophages) were performed under a protocol approved by the Institutional Animal Use and Care Committee at Università Cattolica del S. Cuore, Rome, Italy (Permit number: N21, 12/05/2010) and authorized by the Italian Ministry of Health, according to Legislative Decree 116/92, which implemented the European Directive 86/609/EEC on laboratory animal protection in Italy. Animal welfare was routinely checked by veterinarians of the Service for Animal Welfare.

### Bacterial Strains, Plasmids and Culture Conditions

Table 1 lists the bacterial strains and plasmids used in this study. The *R. equi* 103 strain used is devoid of its virulence plasmid. Wild type (WT) or mutant *R. equi* strains and the *Escherichia coli* strain used to construct mutants were routinely grown at 37°C in Brain Heart Infusion (BHI) broth and Luria-Bertani media respectively, with vigorous shaking (200 rpm). When required, antibiotics were added to culture media at the following concentrations: apramycin (30 µg/ml); ampicillin (100 µg/ml) [33]. Dilutions were performed in physiological solution (0.9% NaCl).

**Table 1:** Bacterial strains and plasmids.

Bacterial species and plasmids	Bacterial strains and plasmid names	Relevant characteristics	Source or reference
***R. equi***	103^−^	plasmid-less wild-type strain	[38]
	Δ*katA*	103^−^ isogenic derivative REQ4750 deletion mutant	This study
	Δ*katB*	103^−^ isogenic derivative REQ44520 deletion mutant	This study
	Δ*katC*	103^−^ isogenic derivative REQ26870 deletion mutant	This study
	Δ*katD*	103^−^ isogenic derivative REQ26290 deletion mutant	This study
***E.coli***	Top10	*F-mcrA* Δ*(mrr-hsdRMS-mcrBC)* Π*80lacZ*Δ*M15* Δ*lacX74 recA1 araD139 galU* *galK* Δ*(ara-leu)7697 rpsL (Str^r^) endA1 nupG*	Invitrogen
**Plasmids**	pRHE2	pUC19 inserted with *aacC4*	[33]
	pUC19::*katA*	pUC19 inserted with *katA*	This study
	pUC19::*katB*	pUC19 inserted with *katB*	This study
	pUC19::*katC*	pUC19 inserted with *katC*	This study
	pUC19::*katD*	pUC19 inserted with *katD*	This study
	pUC19-Δ*katA*::*aacC4*	pUC19 with Δ*katA*::*aacC4* mutant allele (suicide vector for *katA* mutagenesisby gene replacement)	This study
	pUC19-Δ*katB*::*aacC4*	pUC19 with Δ*katB*::*aacC4* mutant allele (suicide vector for *katB* mutagenesisby gene replacement)	This study
	pUC19-Δ*katC*::*aacC4*	pUC19 with Δ*katC*::*aacC4* mutant allele (suicide vector for *katC* mutagenesisby gene replacement)	This study
	pUC19-Δ*katD*::*aacC4*	pUC19 with Δ*katD*::*aacC4* mutant allele (suicide vector for *katD* mutagenesisby gene replacement)	This study

### Construction of Four Catalase Mutants

A double crossover homologous recombination strategy based on suicide vectors derived from pUC19 containing a cassette consisting of the upstream homologous region, the apramycin resistance gene (*aaC4*) and the downstream homologous region as previously described [33] was used to inactivate the four catalase genes identified in *R. equi*: REQ4750, REQ44520, REQ26870 and REQ26290, respectively named *ΔkatA, ΔkatB, ΔkatC and ΔkatD*. Briefly, for Δ*katA* construction, a DNA fragment consisting of the *katA* gene and ≈600 bp up-and downstream of the start and stop codons was amplified with primers L39 and L79 containing respectively *EcoR*I and *Xba*I restriction sites (Table S1). Then, the product was inserted into pUC19 using *EcoR*I and *Xba*I restriction sites. To delete the *katA* gene’s internal region, an inverse PCR was performed on the resulting pUC19::*katA* plasmid using divergent primers L162 and L163 with *Nsi*I restriction site sequence in order to obtain a linear pUC19-*ΔkatA* plasmid containing a 70% deleted version of *katA*. Separately, the *aacC4* gene was amplified using primers L118 and L119 each containing an *Nsi*I restriction site. Both pUC19-*ΔkatA* and *aacC4* amplicons were digested by *Nsi*I and ligated to obtain a pUC19-*ΔkatA*::*aacC4* plasmid. This plasmid was introduced into *R. equi* by electroporation, and transformants were selected on BHI agar supplemented with 80 µg/ml apramycin. Allelic exchange double recombinants were selected as previously described [33]. The same procedure was applied for the construction of Δ*katB*, Δ*katC* and Δ*katD*. Construction was verified by Southern blot (data not shown). The primers used are listed in Table S1.

### Hydrogen Peroxide Challenge Assays

Ten ml of exponentially growing cultures of *R. equi* WT, Δ*katA*, Δ*katB*, Δ*katC* or Δ*katD* (Optical Density at 600 nm (OD_600_) of 0.2) were harvested by centrifugation and pellets were resuspended in 10 ml of 0.9% (w/v) NaCl. OD_600_ of stationary growing cultures were measured and the volume necessary for obtaining an OD_600_ of 0.2 in a volume of 10 ml was harvested by centrifugation and resuspended in 10 ml of 0.9% (w/v) NaCl. Both cell suspensions were treated with 80 mM H_2_O_2_ (Riedel de Haën) and bacterium viability was determined immediately before treatment and after 30 min of incubation at 37°C, 200 rpm, by viable counts of bacteria made by serially diluting samples and plating onto BHI agar. Colony-Forming Units (CFU) were enumerated after 48 h of incubation at 37°C by counting two plates at two different dilutions. All experiments were performed in triplicate.

### Survival Assays in Mouse Peritoneal Macrophages

The survival of *R. equi* WT, Δ*katA*, Δ*katB*, Δ*katC* and Δ*katD* in mouse peritoneal macrophages was tested by using an *in vivo/in vitro* infection model as described previously [34], [35]. Briefly, after 16 h of growth, bacterial cells were pelleted and resuspended in an adequate volume of phosphate-buffered saline (PBS) for injection. Male BALB/c mice (10 weeks old) were infected with 10^7^ to 10^8^ cells of each strain by intraperitoneal injection. After an 8 h infection period, peritoneal macrophages were collected by a peritoneal wash, centrifuged then suspended in Dulbecco’s modified Eagle’s medium containing 10 mM HEPES, 2 mM glutamine, 10% bovine fetal serum, and 1X nonessential amino acids supplemented with vancomycin (10 µg/ml) and gentamicin (150 µg/ml). The cell suspension was dispensed into 24-well tissue culture plates and incubated at 37°C under 5% CO_2_ for 2 h. Duplicate wells of infected macrophages were lysed with detergent at 24, 48, and 72 h post infection, and lysates were plated on BHI agar to monitor bacterial survival. All experiments were performed in triplicate, and the results were subjected to statistical analysis using one-way analysis of variance with Bonferroni’s correction post test using GraphPad Prism version 5.00 for Windows (GraphPad Software, San Diego, CA).

### Catalase Overexpression Assays

*R. equi* WT in mid-exponential (OD_600_ of 0.4) and stationary (OD_600_ of 1) growth phases was treated with 50 mM H_2_O_2_. Previous results have shown that 50 mM H_2_O_2_ is a sublethal concentration (data not shown). A control for each growth phase was performed without H_2_O_2_ treatment. Bacterial cells were incubated (37°C, 200 rpm) for 5, 10, 20, 30 or 60 min after treatment and total bacterial RNA was isolated: cells were harvested by centrifugation, rinsed in PBS 1X and harvested again by centrifugation. Cells were resuspended in 50 mg/ml lysozyme (Sigma) prepared with Tris/HCl-Ethylenediaminetetraacetic acid (EDTA) buffer, and lysed with a Ribolyser (Hybaid). Samples were incubated for 90 min at 37°C. Total RNA was isolated using the RNeasy RNA mini kit (Qiagen) according to the manufacturer’s instructions. A 15-min on-column DNA digestion was also performed. After elution, RNA was treated with Turbo DNA-free (Ambion) according to the manufacturer’s instructions. The concentrations and quality of total RNA were evaluated using Experion (Biorad) with the RNA Std Sens kit, according to the manufacturer’s instructions. One microgram of total RNA was reverse-transcribed using the Omniscript enzyme (Qiagen), recombinant RNAsin (Promega) and random hexamer primers (Invitrogen) according to the manufacturer’s instructions.

The resulting cDNAs were used for subsequent PCR amplification with specific primers, designed using Primer3 software. Primer sequences are listed in Table S1 and were used with the QuantiFast SYBR Green PCR mix (Qiagen) according to the manufacturer’s instructions, using a Mastercycler® ep *realplex* (Eppendorf). The quantification of 16S RNA levels was used as an internal control. Two independent bacterial cultures were used to obtain two different RNA extracts. Each RNA was reverse-transcribed twice in order to obtain four cDNAs. Amplification, detection and real-time analysis were performed in triplicate with the four cDNAs. The overexpression factor (OF) in treated bacteria *vs* control, was calculated using the 2^−ΔΔCt^ method [36]. To evaluate the efficiency of the amplification, a standard curve was constructed using the cycle threshold (*C_T_*) versus 10-fold dilution (data not shown). The reaction’s specificity is given by detection of the melting temperatures (Tm) of the amplification products *via* the melting curve.

## Supporting Information

Table S1 **Oligonucleotide primers used in this study.**
(DOCX)
