# Supplementary material for: Rhodococcus equi’s Extreme Resistance to Hydrogen Peroxide Is Mainly Conferred by One of Its Four Catalase Genes
Source: PLoS One. 2012 Aug 6;7(8):e42396. doi: 10.1371/journal.pone.0042396 (PMC3412833; doi:10.1371/journal.pone.0042396)
Supplement: Table S1 — Oligonucleotide primers used in this study. (DOCX) [file pone.0042396.s001.docx]

**Table S1. Oligonucleotide primers used in this study**
